# A PilZ domain protein interacts with the transcriptional regulator HinK to regulate type VI secretion system in *Pseudomonas aeruginosa*

**DOI:** 10.1016/j.jbc.2024.105741

**Published:** 2024-02-09

**Authors:** Tianfang Cheng, Qing Wei Cheang, Linghui Xu, Shuo Sheng, Zhaoting Li, Yu Shi, Huiyan Zhang, Li Mei Pang, Ding Xiang Liu, Liang Yang, Zhao-Xun Liang, Junxia Wang

**Affiliations:** 1 Integrative Microbiology Research Centre, Guangdong Province Key Laboratory of Microbial Signals and Disease Control, South China Agricultural University, Guangzhou, China; 2 School of Biological Sciences, Nanyang Technological University, Singapore, Singapore; 3 Key Laboratory of Basic Pharmacology of the Ministry of Education, Joint International Research Laboratory of Ethnomedicine of the Ministry of Education and Key Laboratory of Basic Pharmacology of Guizhou Province, Zunyi Medical University, Zunyi, Guizhou, China

**Keywords:** c-di-GMP, PilZ, TssZ, HinK, PqsR, *Pseudomonas aeruginosa*

## Abstract

Type VI secretion systems (T6SS) are bacterial macromolecular complexes that secrete effectors into target cells or the extracellular environment, leading to the demise of adjacent cells and providing a survival advantage. Although studies have shown that the T6SS in *Pseudomonas aeruginosa* is regulated by the Quorum Sensing system and second messenger c-di-GMP, the underlying molecular mechanism remains largely unknown. In this study, we discovered that the c-di-GMP-binding adaptor protein PA0012 has a repressive effect on the expression of the T6SS HSI-I genes in *P. aeruginosa* PAO1. To probe the mechanism by which PA0012 (renamed TssZ, Type Six Secretion System -associated PilZ protein) regulates the expression of HSI-I genes, we conducted yeast two-hybrid screening and identified HinK, a LasR-type transcriptional regulator, as the binding partner of TssZ. The protein-protein interaction between HinK and TssZ was confirmed through co-immunoprecipitation assays. Further analysis suggested that the HinK-TssZ interaction was weakened at high c-di-GMP concentrations, contrary to the current paradigm wherein c-di-GMP enhances the interaction between PilZ proteins and their partners. Electrophoretic mobility shift assays revealed that the non-c-di-GMP-binding mutant TssZ^R5A/R9A^ interacts directly with HinK and prevents it from binding to the promoter of the quorum-sensing regulator *pqsR*. The functional connection between TssZ and HinK is further supported by observations that TssZ and HinK impact the swarming motility, pyocyanin production, and T6SS-mediated bacterial killing activity of *P. aeruginosa* in a PqsR-dependent manner. Together, these results unveil a novel regulatory mechanism wherein TssZ functions as an inhibitor that interacts with HinK to control gene expression.

*Pseudomonas aeruginosa* is a widespread human opportunistic pathogen, typically associated with nosocomial infections and responsible for many human diseases. This bacterium is a major contributor to chronic lung infections in patients with cystic fibrosis (1). *P. aeruginosa* can adapt to a variety of conditions by altering its global gene expression. A large number of virulence factors harbored in *P. aeruginosa*’s genome play vital roles in adaptation to different environments (2). Virulence factors contribute significantly to infection progression by helping the bacteria avoid damage from the host immune response and facilitate pathogenesis (3).

The Quorum sensing (QS) system modulates several virulence-associated phenotypes in *P. aeruginosa*. For example, swarming motility requires a complex coordination of flagella, type IV pili, and biosurfactants (4). Production of rhamnolipids, key biosurfactants for bacterial surface translocation and reducing surface tension on semisolid surfaces (5, 6, 7), is modulated by the RhlR/RhlI QS system. This system regulates the expression of rhamnolipid-related genes (*rhlABC*) through the upstream QS transcription factor RhlR (8, 9, 10). Another virulence factor, pyocyanin, a redox-active phenazine compound that can damage host tissues and cause cystic fibrosis airway pathogenesis (11) is regulated through its biosynthetic pathway. Gene clusters *phzA1B1C1D1E1F1G1* (*phzA1-G1*) and *phzA2B2C2D2E2F2G2* (*phzA2-G2*) encode a series of enzymes involved in the biosynthesis of the pyocyanin precursor phenazine-1-carboxylic acid (PCA). PCA is ultimately converted to pyocyanin by PzhM and PhzS (12). Notably while PqsR, through the synthesis of QS quinolones, is the main regulator of pyocyanin production, the LasR/LasI and RhlR/RhlI systems also exert a degree of control (13).

The type VI secretion system (T6SS) is an important protein nanomachine found in numerous Gram-negative bacteria and serves to translocate effector proteins to both prokaryotic and eukaryotic cells. *P. aeruginosa* utilizes three such T6SSs, designated HSI-I (or H1-T6SS), HSI-II (or H2-T6SS), and HSI-III (or H3-T6SS), to enhance its competitive advantage (14). HSI-I primarily delivers toxic effectors into target cells to facilitate bacterial community competition (15). HSI-II and HSI-III, however, exhibit broader functionality and translocate protein effectors into both prokaryotic and eukaryotic cells, leading to either growth inhibition or death of target cells (16).

HSI-I expression in *P. aeruginosa* is subject to multi-layered control across transcriptional, post-transcriptional, and post-translational levels (17). At the transcriptional level, the QS system exerts control over the HSI-I gene cluster. LasR and PqsR act as indirect negative regulators (18) while PqsE directly represses HSI-I expression (19). AmrZ demonstrates contrasting regulatory roles, it is a positive regulator of HSI-I expression through direct binding to the promoter region of HSI-I in *P. aeruginosa* PA14 (20). Conversely, in *P. aeruginosa* PAO1, AmrZ becomes a negative regulator by inducing cyclic-3′5′-diguanylic acid (c-di-GMP) synthesis. C-di-GMP, in turn, binds FleQ to inhibit the expression of HSI-I (21). Additionally, the GacS/GacA pathway, controlled by the RetS and LadS systems, also contributes to HSI-I regulation. RetS represses the GacS/GacA pathway while LadS positively controls it to affect HSI-I expression (22, 23, 24).

C-di-GMP, a ubiquitous bacteria second messenger, controls diverse processes, including bacterial motility-sessility switch and acute-chronic infection transition, and participates in complex physiological responses (25). First discovered in 1987 as an allosteric activator of bacterial cellulose synthase in *Gluconacetobacter xylinus* (formerly *Acetobacter xylinum*) (26), subsequent studies revealed its broad biological role across the bacterial signaling network. The synthesis and degradation of c-di-GMP are regulated by two enzyme families: diguanylate cyclases (DGCs) and phosphodiesterases (PDEs). GGDEF domains within DGCs are responsible for catalyzing c-di-GMP production from two molecules of GTP, while the EAL or HD-GYP domains in PDEs catalyze its degradation of c-di-GMP (27, 28, 29). C-di-GMP mediates its regulatory function through two main modes: (1) influencing specific gene expression to coordinate cellular responses, and (2) acting as a common protein allosteric regulator to control various biological processes (30). In *P. aeruginosa*, effectors of c-di-GMP, including proteins and RNA riboswitches, play essential roles in mediating this signaling pathway (31). Specialized domains like the PilZ domain or degenerate GGDEF and EAL domains are present in these protein effectors (32). The *P*. *aeruginosa* genome encodes eight PilZ domain-containing proteins, including five single-domain PilZ proteins. While the functions of PA2960 (PilZ), PA2799 (HapZ), and PA4608 (MapZ) have been elucidated (33, 34, 35), the functions of PA0012 and PA4324 remain unknown. PA0012 have been reported to bind c-di-GMP with high affinity, confirming its role as c-di-GMP receptor (36, 37, 38). However, the potential involvement of these PilZ proteins in the regulation of HSI-I expression remains unexplored.

This study identifies PA0012 as a previously unknown repressor of the HSI-I gene cluster in *P. aeruginosa*. We conducted transcriptomic analysis on a *P. aeruginosa* PAO1 strain with *PA0012* deleted (Δ*PA0012*) and its corresponding complemented strain (Δ*PA0012*^*C*^) and discovered that PA0012 negatively regulates HSI-I genes. Following that, through yeast two-hybrid screening, we uncovered HinK, a LasR-type transcriptional regulator, as a protein partner of PA0012. Our results indicate that HinK physically interacts with PA0012 to regulate the expression of PqsR, HSI-I, and various virulence-associated traits in *P. aeruginosa*. These findings unveil the cellular function of a c-di-GMP-binding adaptor and a new transcriptional regulatory circuit that further expands our understanding of c-di-GMP’s role in *P. aeruginosa*.

## Results

### PA0012 negatively regulates the expression of HSI-I genes in *P. aeruginosa*

**Figure 1:**
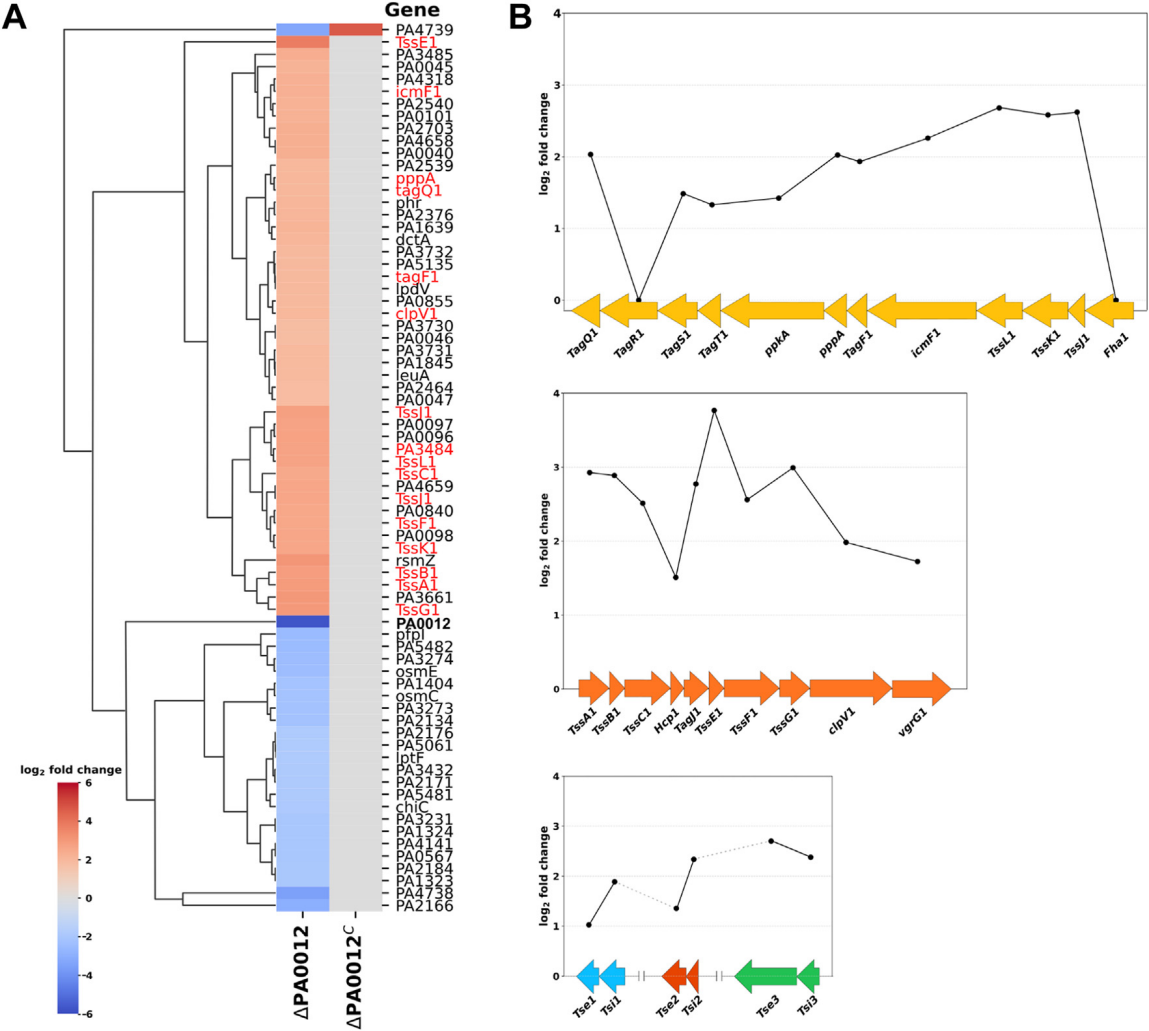
**Deletion of *PA0012* increases expression of the Type VI secretion system HSI-I gene cluster.***A*, heatmap of transcriptional changes in the representative genes of the mutant strain Δ*PA0012*/p vs its complement strain Δ*PA0012*/Δ*PA0012*^*C*^. Genes from the Protein secretion/export apparatus function class are shown in *red* font. *B*, changes in the expression of genes in the HSI-I gene cluster and the secreted toxin protein clusters in the Δ*PA0012* mutant.

The PilZ proteins serve as key c-di-GMP receptors in *P. aeruginosa*, modulating diverse bacterial behaviors at the transcriptional or translational levels (39). However, the functions of the single domain PilZ proteins, PA0012 and PA4324, remain largely uncharacterized. We focused on unraveling the cellular function of PA0012 in this study. To elucidate transcriptional pathways regulated by PA0012, we conducted a transcriptomic analysis using RNA sequencing on Δ*PA0012* and Δ*PA0012*^*C*^ PAO1 strains. Using a log2 fold-change cutoff value of ±1, we identified 72 differentially expressed genes (DEGs) in the Δ*PA0012* strain (Fig. 1*A*). Functional classification of these genes using PseudoCAP revealed that the predominant category of DEGs pertained to “protein secretion and export apparatus”. Notably, the Hcp Secretion Island I (HSI-I) gene cluster was among the highly upregulated genes in Δ*PA0012* (Fig. 1, *A* and *B*), suggesting PA0012’s role as a repressor of HSI-I genes.

To further substantiate the hypothesis that PA0012 could be a negative regulator of the HSI-I cluster, we performed quantitative real-time PCR with cDNA from PA0012-overexpressing and PAO1 strains. The results showed that overexpression of PA0012 resulted in approximately a two-fold downregulation of *tssB1*, *tse2*, *tse3*, *hcp1*, and *tssL1* genes belonging to T6SS HSI-I gene cluster (Fig. S1*A*). We subsequently investigated this effect at the protein level through western blot assays and observed significantly decreased TssB1 expression in a PA0012-overexpressing PAO1 strain where *tssB1* was replaced with HA-tagged *tssB1* at its native chromosomal loci in PAO1 (Fig. S1*B*). Likewise, an increased expression of GFP-tagged ClpV1 was observed in the Δ*PA0012* strain which was successfully complemented by PA0012 overexpression (Fig. S1*C*). These experiments confirm the suppressive effects of PA0012 on the expression of the HSI-I gene cluster and validate the transcriptome experiments. Consequently, PA0012 was renamed as TssZ (T6SS-associated PilZ).

### Identification of the transcriptional regulator HinK as an interacting protein of TssZ by yeast two-hybrid screening

**Figure 2:**
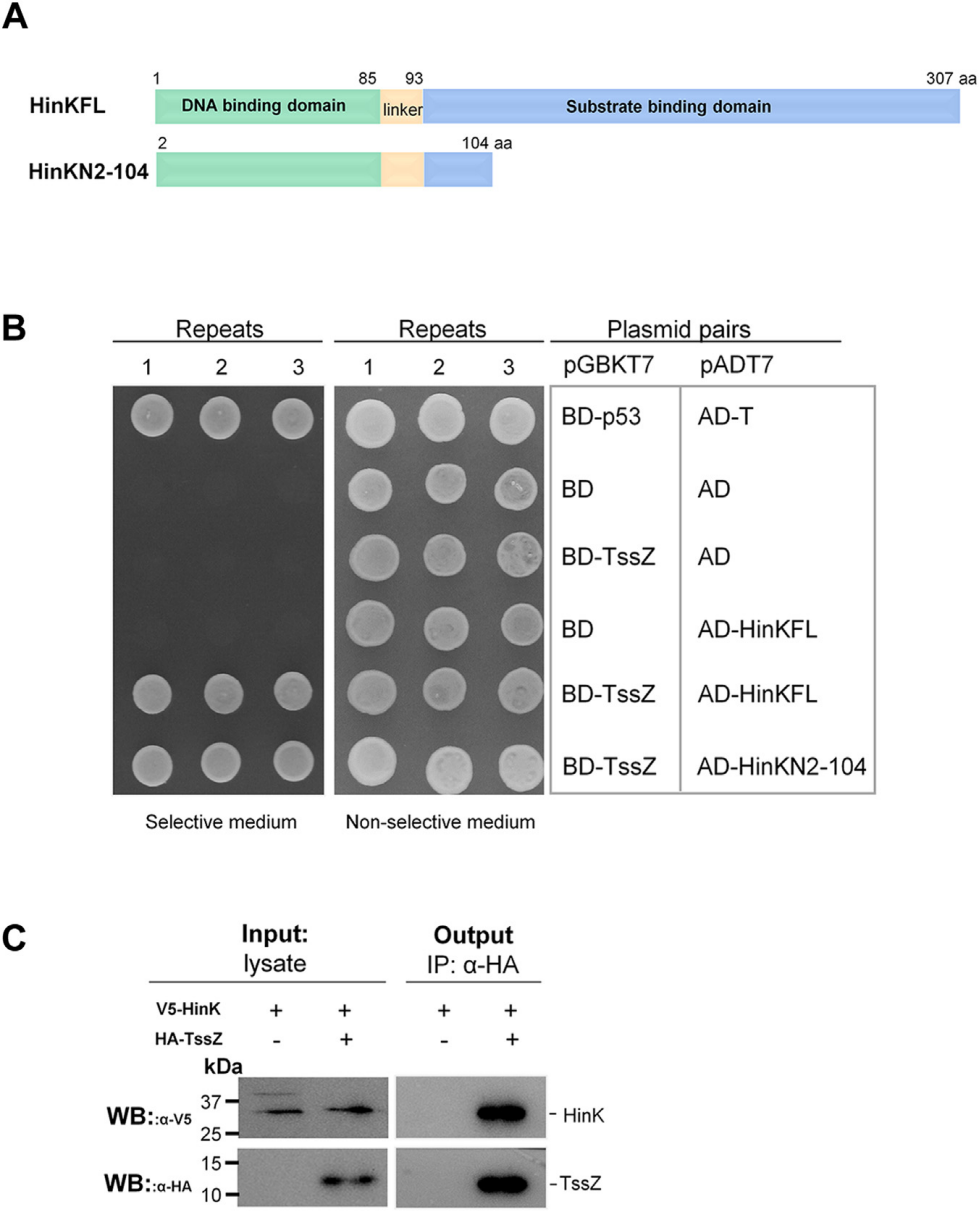
**HinK interacts with TssZ.***A*, domain organization of the transcriptional regulator HinK. HinKFL refers to full length HinK and HinKN2-104 refers to the N-terminal 103 amino acids of HinK. *B*, yeast two-hybrid assay. TssZ was fused to GAL4 DNA-binding domain (BD) and HinK was fused to GAL4 activation domain (AD), respectively. The interaction between HinK and TssZ was tested on selective media. AD-T/BD-p53 was the positive control while AD/BD was the negative control. *C*, co-immunoprecipitation (co-IP) of HinK and TssZ. co-IP assay was performed by incubating cell lysate and Anti-HA Affinity Gel. The precipitated proteins and an aliquot of the cell lysate were visualized by using western blotting with anti-V5 antibody and anti-HA antibody.

The single-domain PilZ proteins, which lack other functional domains have been experimentally shown to function as c-di-GMP receptors by interacting with protein partners (40). Although we successfully identified the protein partners for the PilZ proteins HapZ and MapZ (34, 35), attempts to identify TssZ’s interaction partner using the same approach yielded negative results. Suspecting that endogenous c-di-GMP may weaken rather than enhance the interaction between TssZ and its partner, we turned to yeast two-hybrid assays to leverage the c-di-GMP-free environment in yeast. The yeast two-hybrid screening was conducted using the Matchmaker Gold Yeast Two-Hybrid System, and the bait plasmid pGBKT7-TssZ used in this screening was validated to have no self-activation in the reporter yeast strain. A *P. aeruginosa* genomic DNA library was constructed by using pGADT7 vector and introduced into yeast cells expressing the GAL4-fused TssZ bait protein to probe for potential bait-binding proteins. This effort led to the identification of a prey plasmid containing nucleotides 246,557-246,865 in the *P. aeruginosa* PAO1 genome. This region encodes 103 amino acids in the N-terminal region of the protein HinK (HinKN_2-104_) fused in-frame with the B42 transcriptional activation domain. HinK, a transcriptional regulator, consists of an amino-terminal DNA-binding domain (1–85 aa) and carboxyl-terminal substrate-binding domain (93–307 aa), separated by an acidic linker (Fig. 2*A*). Co-expression of the library HinKN_2-104_ plasmid with TssZ demonstrated their interaction. Specific yeast two-hybrid assay also confirmed the strong interaction between full-length HinK (HinKFL) and the bait TssZ, as evidenced by the robust growth of the yeast colonies harboring the plasmid pair on SD selective medium (Fig. 2*B*). Importantly, these results not only indicate a direct interaction between TssZ and HinK but also reveal that the TssZ-HinK interaction is not c-di-GMP-dependent, as yeast cells lack this molecule. This finding is in contrast with studies on other PilZ domain proteins such as HapZ and MapZ whose interaction with their protein partner is enhanced by c-di-GMP (34, 35).

### The interaction between TssZ and HinK is enhanced at low c-di-GMP levels

To validate the interaction between TssZ and HinK and examine the effect of c-di-GMP on this interaction, we constructed the pBBR1-V5-HinK-TssZ-HA plasmid. This plasmid allows the simultaneous expression of TssZ fused with a HA tag at its carboxyl-terminal (TssZ-HA) and the HinK fused with V5 tag at its amino-terminal (V5-HinK) for co-immunoprecipitation (co-IP) assays. Immunoprecipitation using anti-HA beads, followed by detection with α-HA and α-V5 antibodies, revealed that TssZ-HA interacted with V5-HinK *ex vivo* (Fig. 2*C*).

**Figure 3:**
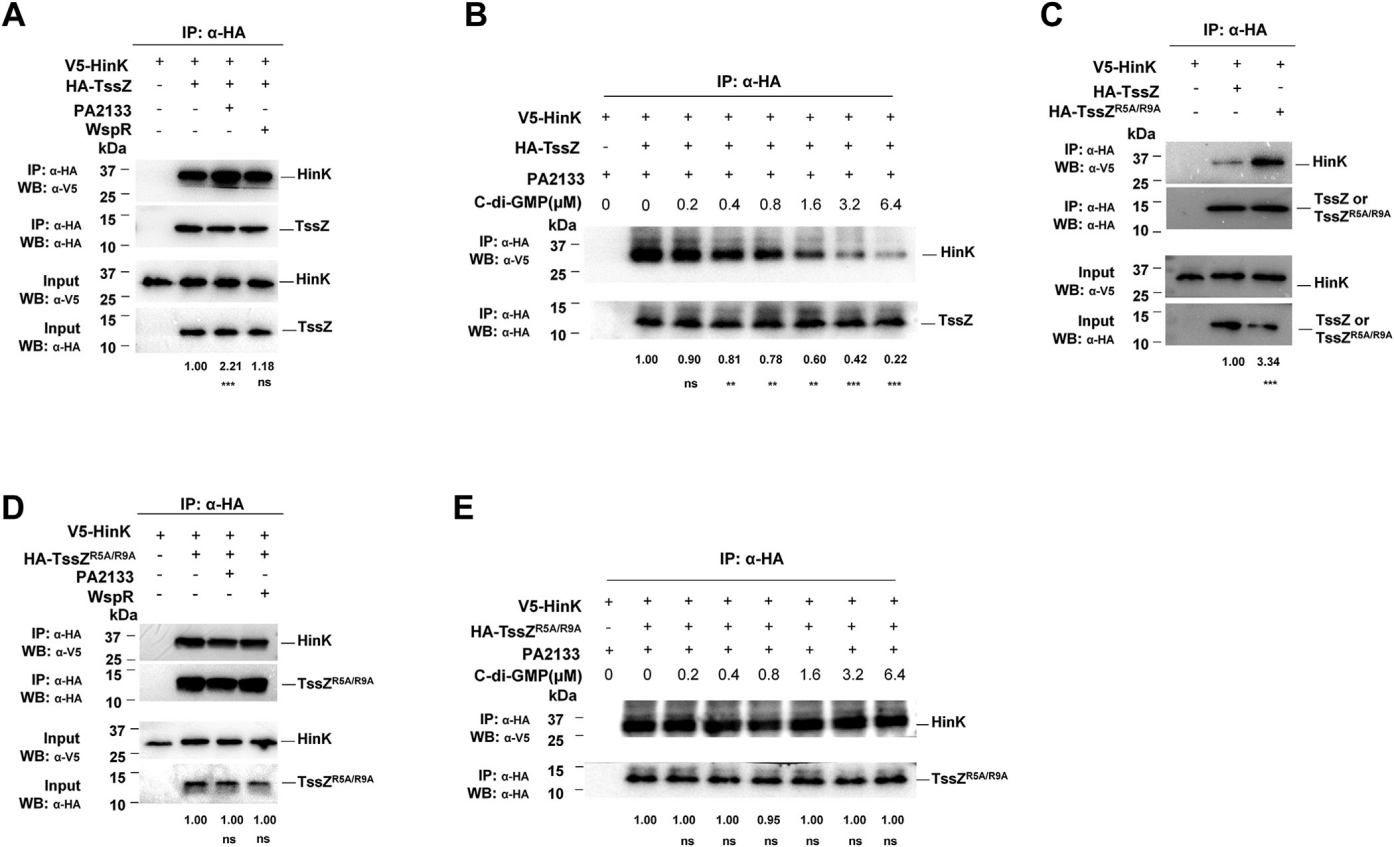
**The interaction between TssZ and HinK is reduced by c-di-GMP.** Co-IP analysis of the interaction between TssZ and HinK under different c-di-GMP concentrations. The intensity of HA-TssZ and V5-Hink bands were determined using ImageJ software and the ratio of HA-TssZ to V5-Hink was calculated and displayed at the bottom of each sample’s respective lane. The ratio for the control strain was used as a baseline of 1 and other ratios were normalized relative to it in all experiments. *A*, co-IP analysis of the interaction between TssZ with HinK at different intracellular concentrations of c-di-GMP. Low and high intracellular c-di-GMP was achieved by the overexpression of the phosphodiesterase PA2133 and the diguanylate cyclase *wspR* respectively. *B*, co-IP assay was used to detect the effect of exogenous c-di-GMP on TssZ-HinK interaction with PA2133 overexpression. 0 μM to 6.4 μM c-di-GMP were added into the immunoprecipitation mixture respectively, following incubation with prewashed beads. The immunoprecipitation result was visualized using western blotting. The PAO1+ pBBR1-V5-HinK-TssZ-HA PA2133 sample without any exogenous nucleotides was used as a control. *C*, co-IP analysis of the interaction between TssZ or TssZ^R5A/R9A^ and HinK. *D*, co-IP analysis of the interaction between TssZ^R5A/R9A^ and HinK in normal, low, and high intracellular levels of c-di-GMP by overexpression of pME6032, pME6032-*PA2133* or pME6032-*wspR*. *E*, co-IP assay was used to detect the effect of exogenous c-di-GMP on TssZ^R5A/R9A^-HinK interaction with PA2133 overexpression. 0 μM to 6.4 μM c-di-GMP were added into the immunoprecipitation mixture respectively, following incubation with prewashed beads. The PAO1+ pBBR1-V5-HinK-TssZ-HA PA2133 sample without any exogenous nucleotides was used as control. All experiments were repeated independently for three times and sample ratios were compared using Student's *t* test. (∗*p* < 0.05, ∗∗*p* < 0.01, ∗∗∗*p* < 0.001, ns: not significant, n = 3).

To further explore the effect of c-di-GMP on TssZ and HinK, we engineered PAO1 strains with a low or high cellular c-di-GMP levels by overexpressing the c-di-GMP phosphodiesterase PA2133 (41) or the diguanylate cyclase WspR (42), respectively. Co-IP experiments revealed a 2.2-fold increase in V5-HinK coimmunoprecipitated with HA-TssZ in the low c-di-GMP cell lysate (PA2133 overexpressing strain) compared to PAO1 control strains, while coimmunoprecipitation was not enhanced in the high c-di-GMP cell lysate (Fig. 3*A*). Given that the TssZ-HinK interaction was not c-di-GMP-dependent, as shown by the yeast two-hybrid assays, we speculated that normal physiological conditions for c-di-GMP levels in cell lysates may have saturated all TssZ-HinK complexes, rendering high cellular c-di-GMP ineffectual. The enhanced interaction between HinK-TssZ in the low c-di-GMP background was gradually diminished in a dose-dependent manner by the addition of exogenous c-di-GMP (0–6.4 μM) (Fig. 3*B*). These results confirmed that the TssZ-HinK interaction is indeed enhanced at low c-di-GMP levels *ex vivo*.

To further test whether TssZ’s c-di-GMP-binding ability is essential for its interaction with HinK. We constructed a plasmid to express and purify a mutant TssZ^R5A/R9A^ protein with arginine (R) to alanine (A) substitutions in its RxxxR motif, rendering it incapable of binding c-di-GMP. Co-IP experiments revealed a 3.3-fold increase in V5-HinK co-immunoprecipitated with TssZ^R5A/R9A^ (Fig. 3*C*). Importantly, the following co-IP assays revealed that the TssZ^R5A/R9A^-HinK interaction remained unaffected by cellular c-di-GMP levels or by the addition of exogenous c-di-GMP (Fig. 3, *D* and *E*). These co-IP results indicate that TssZ^R5A/R9A^, in contrast to TssZ, do not respond to increased c-di-GMP when in a low c-di-GMP background. Together, our observations suggests that the c-di-GMP binding ability of TssZ is required to fine-tune its interaction with HinK in response to altered c-di-GMP levels.

### TssZ^R5A/R9A^ modulates the binding of HinK to the promoter region of quorum sensing regulator PqsR

**Figure 4:**
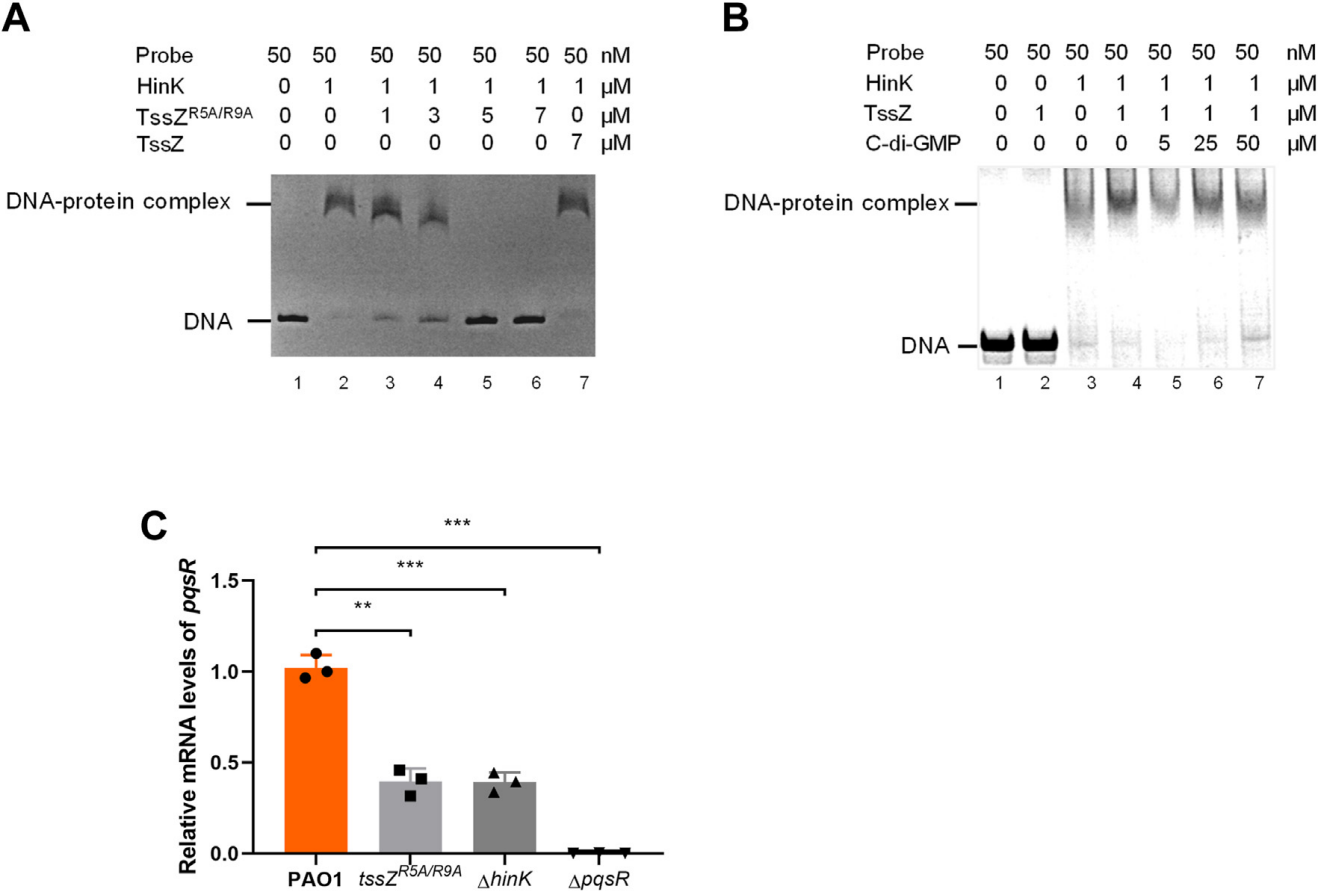
**TssZ**^**R5A/R9A**^**inhibits the binding of HinK to the promoter region of *pqsR* and the transcription of *pqsR*.***A*, TssZ^R5A/R9A^ inhibited the binding of HinK to the promoter of *pqsR* as shown by EMSA. *B*, binding of HinK to the promoter of *pqsR* was not influenced by the interaction between TssZ and c-di-GMP. *C*, transcriptional levels of the *pqsR* gene in PAO1, *tssZ*^*R5A/R9A*^ point mutant, Δ*hinK* mutant and Δ*pqsR* mutant were determined by qRT-PCR, and the *recA* gene was chosen as an internal reference. All experiments were repeated independently for three times and data are represented as the mean ± SD (n = 3). Student's *t* test was used to compare the mRNA levels of the mutants and control (∗*p* < 0.05, ∗∗*p* < 0.01, ∗∗∗*p* < 0.001, n = 3).

HinK is a LysR-type transcriptional regulator (LTTR) and LTTR-dependent transcriptional regulation often requires a co-inducer for transcriptional control of downstream genes (43). It specifically recognizes and binds to the LTTR box (consensus sequence T-N11-A) in the promoter region of *pvdS* and *pqsA*, thereby regulating PQS-related gene expression (44). The consensus sequence, T-N11-A, is a 13-base sequence, consisting of a thymine (T), followed by 11 random bases (N) and ending with an adenine (A). Previous studies have implicated PqsR and its downstream transcription-associated protein PqsE as negative regulators of HSI-I expression (19, 45) and three LTTR boxes were predicted at bases −416 to −374 upstream of the pqsR start codon. Using electrophoretic mobility shift assays (EMSA), we investigated HinK binding sites in the *pqsR* promoter region and identified a span of 334 bases containing an LTTR box that is recognized by HinK (Fig. S2). The direct interaction between TssZ^R5A/R9A^ and HinK raises the possibility of TssZ^R5A/R9A^ influencing the binding activity of HinK to this promoter. To test this hypothesis, we conducted EMSA using TssZ^R5A/R9A^-6× His protein. The effect of TssZ^R5A/R9A^ on HinK binding to the *pqsR* promoter was visualized using native polyacrylamide gel stained with SYBR Safe stain. Results indicated a TssZ^R5A/R9A^-dependent decrease in HinK-bound DNA^PqsR^ probe from 1 to 7 μM, with no detectable bound at 5 to 7 μM. In contrast, the TssZ control showed no inhibition on the HinK-bound DNA^PqsR^ (Fig. 4*A*). Additionally, exogenous c-di-GMP did not affect the formation of TssZ-HinK-DNA^PqsR^ probe complexes in EMSA (Fig. 4*B*). These results demonstrate that TssZ^R5A/R9A^ directly interacts with HinK, preventing its binding to the *pqsR* promoter region.

To ascertain whether the TssZ-HinK complex influences *pqsR* expression *in vivo*, qRT-PCR was used to measure *pqsR* mRNA levels in wild-type PAO1, *tssZ*^*R5A/R9A*^, Δ*hinK*, and Δ*pqsR* mutant strains (all mutant strains were verified by DNA sequencing and qRT-PCR, Fig. S3, *A*–*C*). Bacteria were cultured to an optical density at 600 nm (OD_600_) of 1.0 in LP medium before mRNA extraction. The qRT-PCR results demonstrated lower *pqsR* expression in all three mutants compared to the wild-type PAO1 strain (Fig. 4*C*). These results indicate that TssZ^R5A/R9A^ represses the transcription of the QS regulator *pqsR*, by inhibiting the binding of HinK to the *pqsR* promoter.

### TssZ^R5A/R9A^ and HinK impact swarming motility and pyocyanin production in a *pqsR*-dependent manner

**Figure 5:**
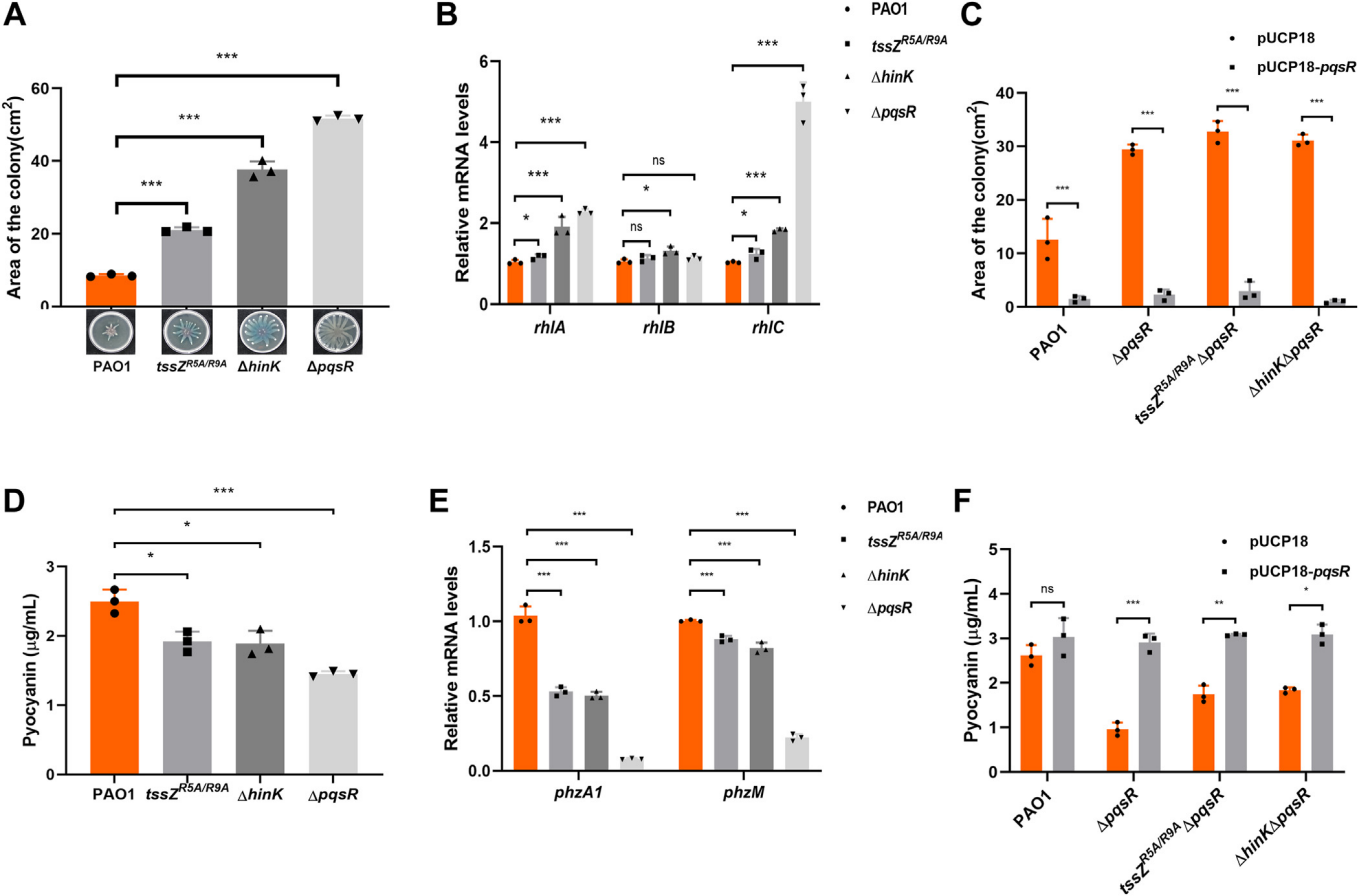
**TssZ**^**R5A/R9A**^**and HinK impact swarming motility and pyocyanin production of *P. aeruginosa* in a PqsR-dependent manner.***A*, swarming motility of the wild-type PAO1, *tssZ*^*R5A/R9A*^ point mutant, Δ*hinK* mutant and Δ*pqsR* mutant strains. Swarming plates were incubated for 16 h at 37 °C after inoculation. Areas of the colonies on swarming plates were measured with ImageJ software and data was analyzed using GraphPad Prism 8 software. *B*, transcription level of the *rhlABC* gene in PAO1, *tssZ*^*R5A/R9A*^ mutant, Δ*hinK* mutant and Δ*pqsR* mutant were determined by qRT-PCR. The *recA* gene was used as an internal reference for comparison. *C*, the swarming motility of PAO1, *tssZ*^*R5A/R9A*^Δ*pqsR* double mutant, and Δ*hinK*Δ*pqsR* double mutant strains without/with overexpression of the *pqsR* gene. *D*, the production of pyocyanin of *P. aeruginosa* strains in LP medium. *E*, transcription levels of the *pzhA1*, and *pzhM* in PAO1, *tssZ*^*R5A/R9A*^ mutant, Δ*hinK* mutant and Δ*pqsR* mutant were determined by qRT-PCR. *F*, the pyocyanin production of PAO1, *tssZ*^*R5A/R9A*^Δ*pqsR* double mutant, and Δ*hinK*Δ*pqsR* double mutant strains without/with overexpression of the *pqsR* gene. All experiments were repeated independently for three times and data are represented as the mean ± SD (n = 3). (∗*p* < 0.05, ∗∗*p* < 0.01, ∗∗∗*p* < 0.001, n = 3).

A recent study has highlighted the role of HinK in regulating QS-related virulence traits in PAO1 (44). The TssZ-HinK-PqsR relationship discovered so far implies that TssZ is likely involved in the regulation of QS-related virulence traits. We, therefore, investigated two phenotypes under QS control, swarming motility and pyocyanin production (46, 47), in *tssZ*^*R5A/R9A*^, Δ*hinK*, and Δ*pqsR* mutant strains. As assessed by colony area on a semi-solid surface (48), the Δ*hinK*, *tssZ*^*R5A/R9A*^, and Δ*pqsR* strains displayed increased swarming motility in comparison to the wild-type PAO1 (Fig. 5*A*). qRT-PCR assays also showed a concordant increase in transcriptional levels of the *rhlA* and *rhlC* genes compared to the wild-type strain PAO1 (Fig. 5*B*). The *rhlABC* gene encodes enzymes participating in the biosynthesis of the biosurfactant rhamnolipid, which is essential for the swarming motility of *Pseudomonas aerugonisa*. We further observed that the expression of *pqsR* restored the swarming motility of Δ*pqsR*, *tssZ*^*R5A/R9A*^Δ*pqsR, and* Δ*hinK*Δ*pqsR* strains to wild-type levels (Fig. 5*C*). These results indicate that TssZ and HinK influence swarming motility through transcriptional regulation of the *rhlABC* gene cluster expression in a *pqsR*-dependent manner.

Pyocyanin biosynthesis, associated with the PQS system, requires the *phzA*-*phzG* gene cluster that is responsible for phenazine production in *P. aeruginosa*. *phzM* and *phzS* encode two phenazine-modifying enzymes that convert phenazine to pyocyanin (12). We observed that the pyocyanin production of the *tssZ*^*R5A/R9A*^ and Δ*hinK* strains were significantly reduced compared with that of the wild-type PAO1, mirroring the phenotype of the Δ*pqsR* mutant strain (Fig. 5*D*). Correspondingly, downregulation of the *pzhA1* and *pzhM* genes in these three mutants were observed (Fig. 5*E*). Furthermore, *pqsR* expression *via* the pUCP18-*pqsR* plasmid restored the pyocyanin production in these mutants to wild-type levels (Fig. 5*F*). These findings indicate that TssZ and HinK influence pyocyanin production through *pqsR*.

### TssZ^R5A/R9A^ and HinK exert control over bacterial killing activity by modulating HSI-I gene expression in a *pqsR-*dependent manner

**Figure 6:**
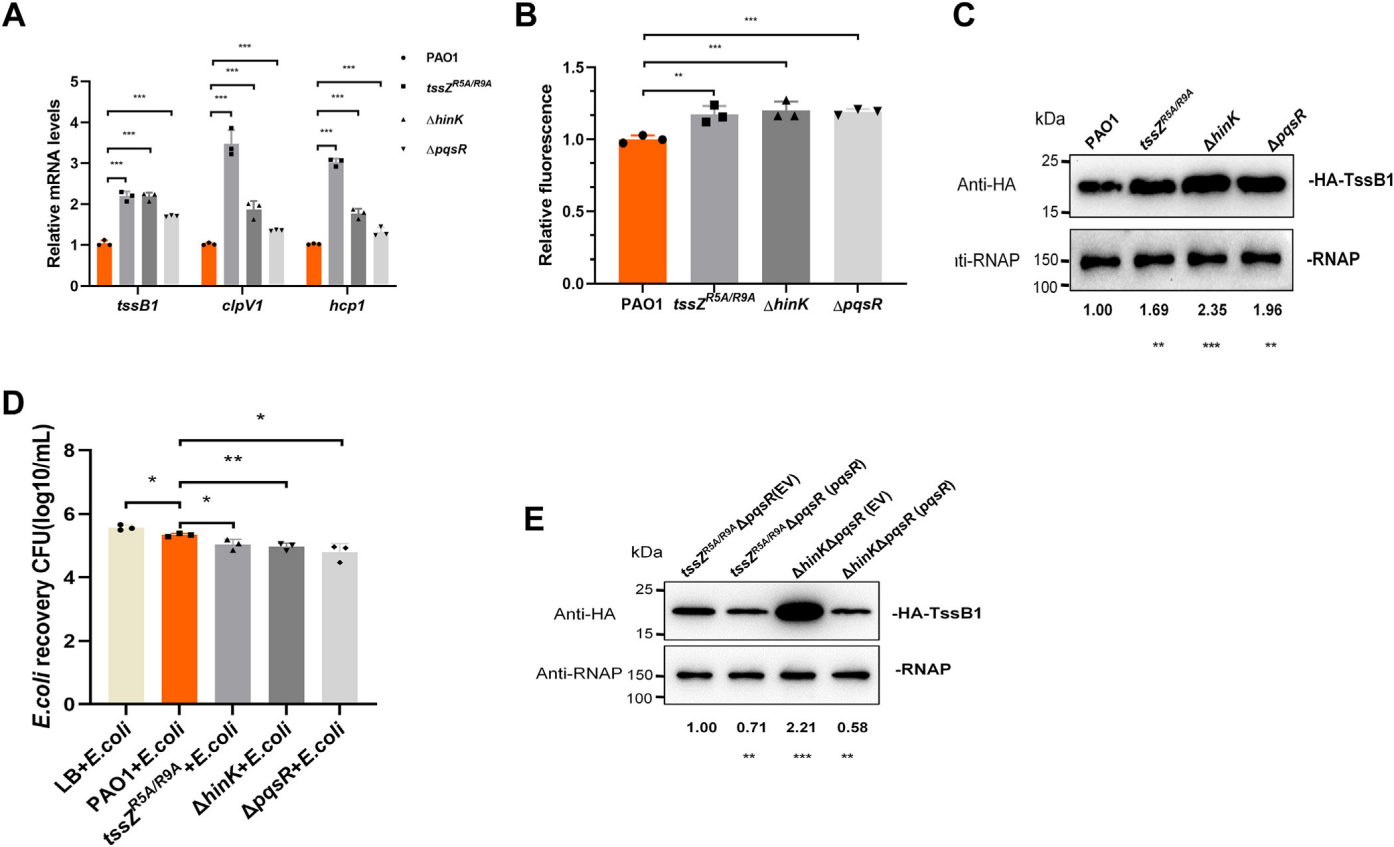
**TssZ**^**R5A/R9A**^**and HinK regulate bacterial killing activity by mediating HSI-I gene expression.***A*, transcription levels of the *tssB1*, *clpV1* and *hcp1* in wild type PAO1, *tssZ*^*R5A/R9A*^ point mutant, Δ*hinK* mutant and Δ*pqsR* mutant strains. The *recA* gene was chosen as an internal reference. *B*, GFP expression is driven by the promoter of *tssA1* in different genetic backgrounds as measured by relative fluorescence. *C*, the expression levels of TssB1-HA were detected in the wild-type PAO1, *tssZ*^*R5A/R9A*^, Δ*hinK* and Δ*pqsR* strains by western blotting. The intensity of bands were measured using ImageJ software. The relative intensity ratio of the TssB1-HA band to the RNAP band for each strain are indicated at the *bottom* of their respective lanes. The intensity ratio of the control PAO1 strain is defined as 1 and other ratios are normalized relative to it. *D*, bacterial killing assays of *P. aeruginosa* to *E. coli* DH5α. *P. aeruginosa* and *E. coli* were mixed at a ratio of 5:1, incubated for 5 h at 37 °C, resuspended in LB, and plated on LB agar plates with 1 mM IPTG, 40 μg/ml X-gal and 50 μg/ml apramycin. *E*, the expression levels of TssB1-HA protein were detected in the *tssZ*^*R5A/R9A*^Δ*pqsR*, Δ*hinK*Δ*pqsR* strains with/without *pqsR* overexpression by western blotting. The expression level of RNA polymerase (RNAP) alpha subunit was used as an internal reference and the intensity of bands were measured using ImageJ software. The relative intensity ratio of the TssB1-HA band/RNAP band for each strain are indicated at the bottom of their respective lanes. The intensity ratio of the control *tssZ*^*R5A/R9A*^Δ*pqsR*-pUCP18 strain is defined as 1 and other ratios normalized relative to it. All experiments were repeated independently for three times and data are represented as the mean ± SD (n = 3). Student's *t* test was used to compare each strain and the control strain (∗*p* < 0.05, ∗∗*p* < 0.01, ∗∗∗*p* < 0.001, n = 3).

To examine TssZ and HinK’s role in regulating of HSI-I genes by controlling PqsR expression in *P. aeruginosa*, we studied expression levels of the HSI-I representative genes *tssB1* and *clpV1* in *tssZ*^*R5A/R9A*^, Δ*hinK* and Δ*pqsR* strains. qRT-PCR assays confirmed the upregulation of both genes all three mutant strains (Fig. 6*A*). This finding was further supported by increased green fluorescent protein (GFP) reporter activity driven by the *tssA1-C1* promoter in *tssZ*^*R5A/R9A*^, Δ*hinK* and Δ*pqsR* mutants, as measured using flow cytometry and cell sorting-based GFP-transcriptional fusion assay. A comparison of the average fluorescence of 50,000 cells from each mutant showed increased relative GFP fluorescence intensity was measured in all three mutants (Fig. 6*B*). These findings indicate that TssZ and HinK function as negative regulators of the HSI-I system.

To determine whether the above-mentioned upregulation of HSI-I gene expression is reflected in protein production, we replaced chromosomal *TssB1* with an HA-tagged TssB1 gene in *tssZ*^*R5A/R9A*^, Δ*hinK*, Δ*pqsR*, and PAO1 strains. Anti-HA antibody was then used to monitor their TssB1 production in these strains. The expression of TssB1 protein was observed to be significantly increased in the *tssZ*^*R5A/R9A*^, Δ*hinK*, and Δ*pqsR* mutants, despite varying mRNA levels of TssB1 among these three mutants (Fig. 6*C*). Given that the HSI-I gene cluster of the T6SS system primarily serves an antibacterial function, a bacterial competition assay using *Escherichia coli* prey was used to compare the bacterial killing abilities of the above-mentioned strains. Our results showed that all three mutant strains displayed enhanced killing of *E. coli* when compared to the control PAO1 strain (Fig. 6*D*). Thus, our findings suggest that HinK and TssZ, like PqsR, negatively regulate the HSI-I gene cluster expression and bacterial killing activity.

To further analyze the relationship between the TssZ^R5A/R9A^/HinK complex and PqsR in regulating the expression of HSI-I, we examined the HA-tagged TssB1 expression by overexpressing PqsR in *tssZ*^*R5A/R9A*^Δ*pqsR* and Δ*hinK*Δ*pqsR* mutants. The same strains harboring an empty pUCP18 vector were used as negative controls. We observed that PqsR-overexpression resulted in a significantly decreased TssB1 expression in *tssZ*^*R5A/R9A*^ mutation and *hinK* deletion strains. This indicates that the regulation of HSI-I by TssZ^R5A/R9A^ and HinK is dependent on the expression level of *pqsR* in cells (Fig. 6*E*). In totality, our work established a connection between TssZ, HinK, and PqsR in the HSI-I regulatory pathway.

## Discussion

*P. aeruginosa*, an opportunistic pathogen frequently associated with nosocomial infections, is able to adapt to and survive a variety of conditions. C-di-GMP has emerged as a vital second messenger that plays a pivotal role in enabling *P. aeruginosa*’s response to environmental stimuli. C-di-GMP translates environmental cues into cellular responses such as bacterial motility, cell cycle transition, and virulence expression (49). This molecule also facilitates the transition from the planktonic state to the biofilm state, enabling adaptation to hostile environments (50). Various receptors such as PilZ domain proteins, GGDEF domain proteins, and EAL domain proteins that mediate the regulatory functions of c-di-GMP have been characterized (51). Among these, PilZ domain proteins are the most prevalent c-di-GMP receptors (25) and previous studies have identified key RxxxR and [D/N]xSxxG motifs that are necessary for binding c-di-GMP in the canonical PilZ domain (52, 53).

This study focuses on the single-domain PilZ protein, TssZ, which was previously confirmed as a c-di-GMP receptor (36, 38). Many single-domain PilZ proteins bind to a partner protein to perform its biological functions. For example, the single-domain PilZ protein HapZ works with the histidine kinase SagS to regulate two-component signaling in *P. aeruginosa* (34). MapZ, also a single-domain PilZ protein, directly interacts with a chemotaxis methyltransferase to regulate flagellar motor switching in *P. aeruginosa* (35). Hence, we reasoned that TssZ, being a single domain PilZ protein, likely adopts a similar mode of signaling. Using yeast two-hybrid screening, we identified HinK, a member of the LTTR family, as a binding partner of TssZ. Surprisingly, this interaction proved to be independent of c-di-GMP as both TssZ and its c-di-GMP-binding deficient mutant, TssZ^R5A/R9A^, interacted directly with HinK in subsequent experiments.

By manipulating intracellular c-di-GMP level through the overexpression of PDEs (decrease c-di-GMP) or DGCs (increase c-di-GMP) in *P. aeruginosa* (22), we sought to uncover more insights into the interaction between TssZ and HinK. When in a reduced c-di-GMP cellular background, we found that the TssZ-HinK interaction was enhanced and the addition of exogenous c-di-GMP significantly inhibits this interaction. However, increased cellular c-di-GMP had no impact on this interaction. We believe this phenomenon is due to the saturation of TssZ with c-di-GMP under normal physiological conditions, owing to its high affinity for the molecule. Our findings demonstrate that, unlike the established paradigm where c-di-GMP enhances the interaction of PilZ domain adaptors and their protein partners (34, 35), the interaction between TssZ and HinK is attenuated by c-di-GMP.

HinK has been reported to be involved in regulating histamine metabolism, virulence-related genes and expression of *phnAB* and *pqsABCDE* gene clusters by binding to an LTTR box located on their promoters (44). PqsR and pqsE are transcriptional activators located within the PQS gene cluster and previous studies have also shown that both proteins repress HSI-I expression (19, 45, 54). These reports, when considered together with our finding that TssZ suppresses the expression of the HSI-I genes (Fig. 1*A*), hint that TssZ may indirectly regulate the expression of HSI-I genes *via* HinK and the QS system. We noted that the promoter of *pqsR* harbors three conserved LTTR boxes (Fig. S2*A*) and used EMSA to confirm that HinK indeed binds to this region (Fig. S2*B*). Consequently, we sought to identify the regulatory pathway between TssZ, HinK, and PqsR. We thus examined virulence-associated traits in the *tssZ*^*R5A/R9A*^, *hinK*-deletion, and *pqsR-*deletion mutant strains and found that all three mutants exhibited similar changes to HSI-I expression, rhamnolipid synthesis, swarming motility, and pyocyanin production. These observations provide functional evidence for the relationship between TssZ and HinK *in vivo* and show that TssZ inhibits the activity of HinK. The yeast two-hybrid assay results also suggest that TssZ interacts with the DNA-binding domain of HinK (Fig. 2*A*), preventing it from binding to DNA. HinK was also reported to require imidazole-4-acetic acid, an intermediate product from histamine metabolism, as a cofactor (44). Whether cofactors are involved in the TssZ-HinK regulatory pathway and the exact mechanism of this interaction, warrants further investigation.

**Figure 7:**
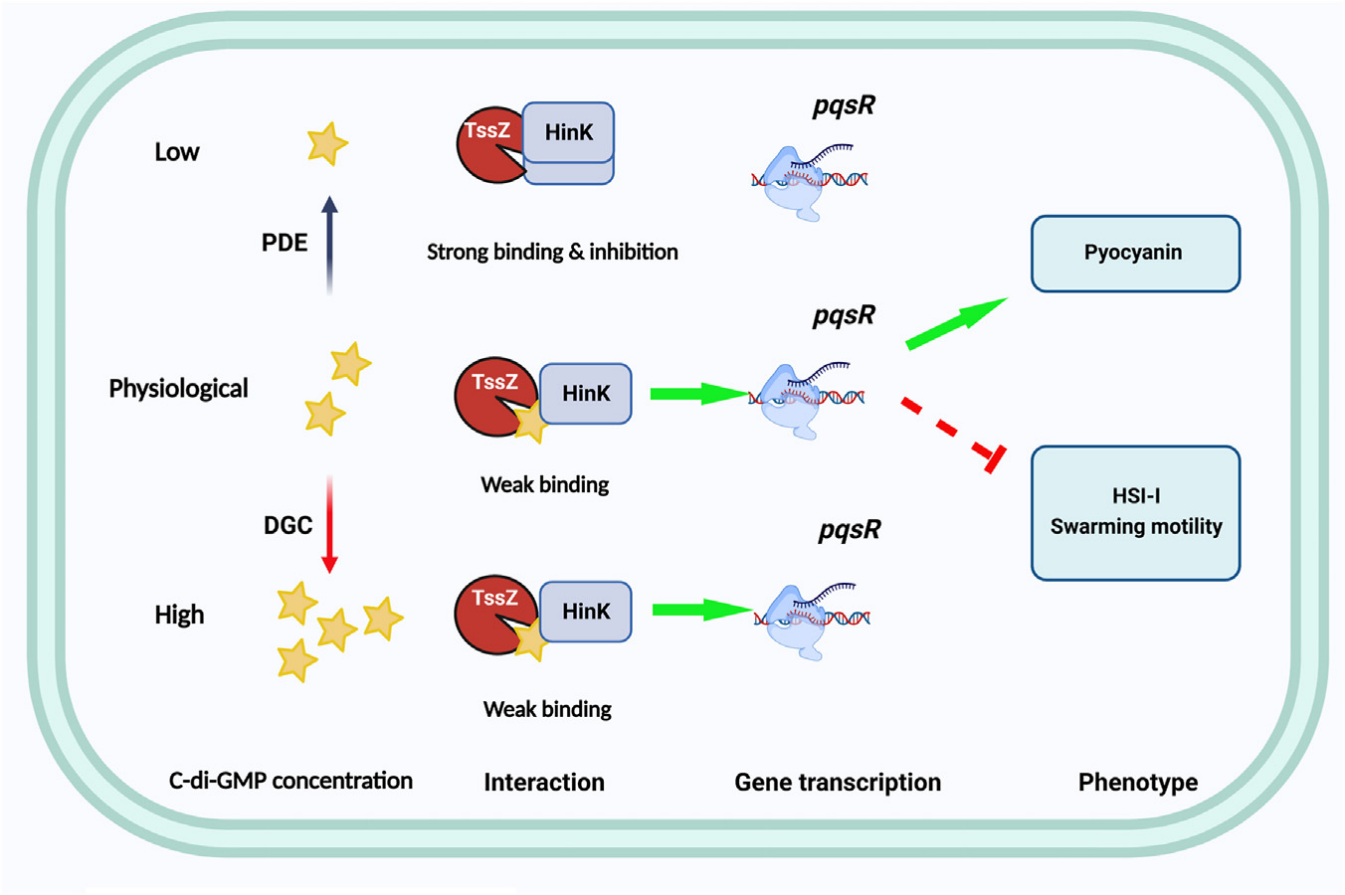
**Proposed mechanistic model of the TssZ/HinK complex in *P. aeruginosa*.***Arrows* and *bars* represent activation or inhibition of expression respectively; solid lines mean a direct regulation or direct connection; *dashed lines* indicate that the regulation mechanism is yet to be determined. In *P. aeruginosa*, HinK positively regulates the transcriptional activity of *pqsR*, which in turn upregulates genes involved in pyocanin production and represses H1-T6SS expression and rhamnolipids biosynthesis. In normal physiological concentrations of c-di-GMP, the receptor TssZ binds to and prevents HinK from binding to DNA, thereby inhibiting the activity of HinK to a certain degree. Decreased intracellular c-di-GMP *increases* the interaction between TssZ and HinK, further *enhancing* the inhibition of HinK and through this indirect inhibition of pqsR, causes the biological responses associated with low c-di-GMP in *P. aeruginosa*. c-di-GMP, cyclic-3′5′-diguanylic acid; DGC, Diguanylate cyclase; H1-T6SS, H1-Type VI secretion system; PDE, Phosphodiesterase.

In light of our findings, we propose a regulatory mechanism that is similar to the RpfR-mediated regulation of Bep exopolysaccharide synthesis (55) (Fig. 7). At low levels of c-di-GMP, the TssZ-HinK interaction is enhanced and HinK is sequestered from activating transcription of *pqsR*. With increasing c-di-GMP levels, this interaction is weakened, allowing HinK to activate *pqsR* transcription; however, this effect plateaus upon normal physiological c-di-GMP levels and the TssZ-HinK interaction is unaffected by further increases in c-di-GMP. In conclusion, the results presented in this manuscript support a novel mechanism by which the c-di-GMP-binding adaptor protein, TssZ physically interacts with the LasR-type transcriptional regulator HinK to control various virulence-related phenotypes in a PqsR-dependent manner. Another notable observation from this study is that the TssZ-HinK interaction is weakened, but not enhanced by c-di-GMP, which is in sharp contrast with the majority of other PilZ adaptor proteins reported (34, 35). This surprising observation suggests that the effect of c-di-GMP on protein–protein interaction cannot be simply generalized.

## Experimental procedures

### Strains, plasmids, and media

The bacterial strains and yeast strains used in this study are listed in Table S1. Bacteria strains were grown in Luria-Bertani (LB) broth or Tryptic Soy Broth (TSB). Cultures were maintained in a 37 °C incubator with shaking at 250 rpm, or on LB agar plates in a standard stationary incubator. LB was purchased from Oxoid (Oxoid Ltd, England). TSB was purchased from Solarbio Company. Plasmids used in this study are listed in Table S2. For specific purposes, plasmids were transformed into *P. aeruginosa* and *E. coli* strains. The appropriate antibiotics were added into the medium to maintain the plasmid in *P. aeruginosa* or *E. coli* strains at the following concentrations: gentamicin, 50 μg/ml; tetracycline, 20 μg/ml; kanamycin, 50 μg/ml. Antibiotics were purchased from Sangon Biotech Co, Ltd. The yeasts were cultured with yeast extract-peptone-dextrose (YPD) medium or synthetic dropout (SD) medium in an incubator at 30 °C. YPD, SD, and Dropout (DO) supplements lacking Leu/Trp or Ade/His/Leu/Trp were purchased from Clontech (Clontech Laboratories, Inc). All plasmids used in this study were constructed by homologous recombination and confirmed by DNA sequencing. The plasmids were kept in the *E. coli* DH5α strains, and stored in a −80 °C refrigerator.

### Mutants strain construction and complementation

Gene deletion and point mutant strains were generated through triparental mating according to a previously described protocol with some modification (56). Upstream and downstream fragments of the target gene were amplified separately using high-fidelity PCR and then inserted into pK18 plasmid together for triparental mating. For mutant complementation, the full length of the gene was cloned and inserted into the vector pUCP18. The resulting plasmid was transformed into the mutant strain by electroporation with the parameters of 2500 V, 2 mm cuvette (Bio-Rad).

### RNA-sequence analysis

The assay was performed as described previously (57). Briefly, single colonies of *PA0012* deletion and complementation strains were inoculated into M9 medium (3.39 g/L Na_2_HPO_4_, 1.5 g/L KH_2_PO_4_, 0.5 g/L NH_4_Cl and 0.25 g/L NaCl dissolved in deionized water) supplemented with 2 mM MgSO_4_, 0.1 mM CaCl_2_, and 0.4% glucose, and grown overnight at 37 °C with shaking at 250 rpm. Cultures were then diluted to an OD_600_ of 0.01 and subcultured in 24-well plates at 37 °C for 7 h and immediately mixed with RNA protect Bacteria reagent (Qiagen). After incubation for 5 min at room temperature, samples were pelleted by centrifugation. After pellets were treated with lysozyme, total RNA was extracted with an RNeasy Mini Purification kit (Qiagen). DNA was eliminated by on-column digestion using RNase-free DNase (Qiagen). Ribosomal rRNAs were removed using the Ribo-Zero Magnetic kit (Epicentre). Gene expression analysis was performed in duplicates by RNA sequencing. The rRNA-depleted RNA was fragmented to 200 to 300 base pairs fragments. cDNA synthesis was followed by end repair and adaptor ligation. The libraries were sequenced using the Illumina Hiseq2000 platform with paired-end protocol and 100 nucleotides read lengths. The sequence reads were assembled and analyzed using RNA-Seq and the expression analysis application of CLC genomics Workbench 6.0 (CLC Bio). The following criteria were used to filter the unique sequence reads: minimum length fraction of 0.9, minimum similarity fraction of 0.8, and maximum number of two mismatches. Data were normalized by calculating the reads per kilobase per million mapped reads for each gene. ANOVA and *t* test were performed on transformed data to identify the genes with significant changes in expression (*p* < 0.05).

### Quantitative reverse transcription polymerase chain reaction assay

*P. aeruginosa* was cultured in 2 ml LB medium with shaking at 220 rpm in a 37 °C incubator for 12 h, and then subcultured in 20 ml LP medium until the OD_600_ value reached 1.0. RNA of *P. aeruginosa* was extracted by the RNeasy Mini Kit (QIAGEN) as described in manufacturer instructions. The quantity and quality of the RNA were determined using a NanoDrop (NanoDrop 2000 Technologies) and agarose gel electrophoresis, respectively. 1.0 μg of total RNA was immediately decontaminated of DNA, and then reverse transcribed into cDNA using the FastKing RT Kit (With gDNase) (Tiangen Biotech). The cDNA was diluted ten times and stored at −20 °C for subsequent experiments. qRT-PCR was performed in 96-well plates. Each reaction in a volume of 20 μl contained 10 ng of cDNA template, 10 μl of 2∗ PowerUp SYBR Green Master Mix (Thermo Fisher Scientific), and 200 nM specific primers. Each reaction was repeated in triplicate, and the housekeeping gene *recA* was used as an internal reference. Next, the reactions were conducted under applied biosystems (Thermo Fisher Scientific) using QuantStudio Real-Time PCR system. The reaction procedure (Fast cycling mode) includes 2 min at 50 °C, 2 min at 95 °C, 40 cycles of 30 s at 95 °C and 30 s at 60 °C. The fold changes typically represent the relative mRNA expression levels of specific genes. All the reactions were performed in triplicates. The gene expression level was calculated using the 2^−ΔΔCt^ method.

### GFP transcriptional fusion assay

The GFP reporter gene assay was conducted as described with some modifications (58). The promoter of *tssA1* gene was cloned based on the features of leader sequence of the *PA0082*-*PA0084* (*tssA1-tssB1-tssC1*) operon (59), and inserted into plasmid pPROBE, producing a pPROBE-*ptssA1-GFP* plasmid. This plasmid was electrotransformed into *P. aeruginosa* strains: PAO1, *tssZ*^*R5A/R9A*^, Δ*hinK* and Δ*pqsR*. Transformants were confirmed by PCR. These bacteria were cultured overnight at 37 °C in 2 ml TSB containing 50 μg/ml gentamicin with shaking at 220 rpm. Overnight cultures were diluted to OD_600_ of 0.4, then incubated the 1:20 dilution in 4 ml TSB containing 50 μg/ml gentamicin at 37 °C for 8 h. Cells were harvested by centrifugation at 12,000 rpm for 1 min, and washed with phosphate-buffered saline (PBS) buffer. Cells were resuspended in PBS buffer at ratio 1:1, then diluted 1:10 in PBS to at least 1 ml in a 2 ml tube. The GFP intensity of diluted cells was detected and recorded by flow cytometry, and 50,000 cells were measured by flow cytometry using a CytoFLEX instrument.

### Yeast two-hybrid assay

Yeast two-hybrid assays were performed as recommended by (34). For yeast two-hybrid screening, the *tssZ* gene was amplified and cloned into the bait plasmid pGBKT7 to get the pGBKT7-TssZ. pGBKT7-TssZ and pGADT7 empty vectors were co-transformed into *Saccharomyces cerevisiae* Y2HGold strain together for self-activation detection. The bait plasmid pGBKT7-TssZ was used to screen the candidate interacting proteins from the cDNA library of *P. aeruginosa* which constructed by Shanghai BioGene Biotech Co, Ltd (Shanghai, China). The plasmids of the positive clones screened from SD/- Trp-Leu-His-Ade deficient plates containing 5 mM 3-amino-1,2,4-triazole (3-AT) were extracted for following PCR detection and sequencing comparison. The candidate gene *hinK* was cloned into the yeast two-hybrid vector pGADT7, as the prey plasmid for yeast two-hybrid analysis. pGBKT7-TssZ and pGADT7-HinK were co-transformed into the Y2HGold yeast strain, and selected on SD/- Trp-Leu-His-Ade containing 5 mM 3AT deficient plates to detect the interaction between these two proteins.

### Co-immunoprecipitation assay

To validate the interaction between TssZ and HinK in *P. aeruginosa*, the co-IP assay was carried out as previously described with some modifications (34). Two plasmids pBBR1-V5-HinK and pBBR1-TssZ-HA-V5-HinK were constructed and transformed into PAO1 strain. The strain harboring plasmid pBBR1-V5-HinK consistently expressed the protein V5-HinK, while the strain with plasmid pBBR1-TssZ-HA-V5-HinK expressed both TssZ-HA and V5-HinK. These bacteria strains were cultured overnight at 37 °C in 5 ml LB containing 50 μg/ml gentamicin with shaking, then sub-cultured in 30 ml LB containing 50 μg/ml gentamicin to OD_600_ of 1.0 at 37 °C; subsequently, the protein expression was induced with 0.01% (m/v) arabinose for 3 h with shaking at 220 rpm in a 37 °C incubator. Cells were harvested by centrifugation (13,000*g* for 10 min, 4 °C), and ultrasonic lysed with 5 ml ice pre-cold cell lysis buffer [50 mM *Tris*-HCl (pH 8.0), 150 mM NaCl, 0.05% (v/v) TWEEN-20, and 1:100 (v/v) protease inhibitor PMSF] by an Ultrasonic breaker instrument (GM3200, WIGGRNS) for 20 min until the cells were completely lysed. Cell lysates were clarified by centrifugation at 15,000*g* for 10 min three times. Some of the supernatants were collected as the input sample. The EZview Red Anti-HA Affinity Gel (Sigma-Aldrich) was pre-washed with lysis buffer and collected at 1000*g* for 1 min at 4 °C. For each immunoprecipitation sample, 800 μl of lysate was mixed with 15 μl of the pre-washed beads and incubated for 2 h at 4 °C with slow rotation. The beads were collected by centrifugation and gently washed six times with lysis buffer at 4 °C, and then the immunoprecipitated proteins were eluted with 20 μl elute buffer [100 mM *Tris*-HCl (pH 7.5), 4% (m/v) sodium dodecyl sulphate (SDS)]. The SDS- polyacrylamide gel electrophoresis (PAGE) loading buffer was added into the eluted immunoprecipitated sample, and the samples were boiled at 100 °C for 10 min. Samples were subjected to SDS-PAGE gel and performed western blotting with the HA antibody (Abcam) and V5 antibody (Abcam). Signals were detected with the Clarity Western ECL Substrate (Bio-Rad, Hercules) and images were captured with the Analytik-jena imaging system (Analytik-jena).

### Western blot analysis

To analyze the expression level of HSI-I, HA-tagged TssB1 was knocked into the genome of *P. aeruginosa* replacing the original TssB1. In order to standardize the TssB1-HA expression level, RNA polymerase II (RNAP) was selected as an internal reference for western blot analysis. Strains were cultured overnight in 3 ml TSB with shaking in a 37 °C incubator, then a 1:100 dilution was grown in 20 ml TSB at 37 °C to OD_600_ of 1.0. Cells were harvested by centrifugation (5000 rpm for 10 min, 4 °C), and ultrasonic lysed with 15 ml ice pre-cold cell lysis buffer (50 mM NaH_2_PO_4_, 300 mM NaCl, 10 mM imidazole, 1 mM PMSF pH 8.0) for 15 min until cells were completely lysed. 300 μl cell lysates were collected and mixed with 100 μl the 4× SDS-PAGE sample loading buffer. The mixtures were boiled at 100 °C for 10 min, subjected to SDS-PAGE gel, and electroblotted onto a polyvinylidene difluoride membrane (Amersham BiosciencesUK). The membranes were blotted with the HA antibody (Abcam) (1:2000 dilutions) or RNAP antibody (Abcam) (1:2000 dilutions), followed by the Peroxidase-conjugated AffiniPure Goat Anti-Rabbit IgG (Proteintech) (1:50,000 dilutions). Signals were detected with the Clarity Western ECL Substrate (Bio-Rad, Hercules, CA, USA), and images were captured with the Analytik-jena imaging system. Band intensities were quantified using the ImageJ software (NIH, Bethesda, MD, USA).

### Protein purification

Plasmids were designed to express HinK, TssZ, and TssZ^R5A/R9A^ proteins containing a 6× His tag at C-terminus. The gene fragments were amplified from PAO1 genomic DNA using the primers containing plasmid homologous sequences, and cloned into the pET28a (+) vector. These plasmids were transformed into *E. coli* BL21 (DE3) strain, respectively. *E. coli* BL21 (DE3) strains containing recombinant plasmids were cultured in LB medium supplemented with 50 μg/ml kanamycin at 37 °C overnight with shaking at 250 rpm, 1:1000 dilution was inoculated into 1 L LB containing 50 μg/ml kanamycin at 37 °C with continuous shaking at 250 rpm, to OD_600_ of 0.6. Then, 0.5 mM isopropyl β-D-1-thiogalactopyranoside (IPTG) was added into cultures to induce protein expression at 16 °C with continuous shaking for 20 h. Cell pellets were collected by centrifugation (5000 rpm, 15 min, 4 °C), re-suspended in 50 ml binding buffer (50 mM NaH_2_PO_4_, 300 mM NaCl, 10 mM imidazole, 1 mM PMSF, pH 8.0), lysed by high-pressure homogenizer, and then centrifugation at 15,000 rpm for 60 min at 4 °C. The supernatant was filtered through a 0.45-μm filter, then incubated with 1 ml Ni NTA Magarose beads (SMART LIFESCIENCES, China) for overnight at 4 °C. The beads were collected by gravity flow, added 6 ml wash buffer (50 mM NaH_2_PO_4_, 300 mM NaCl, 20 mM imidazole, 1 mM PMSF, pH 8.0), mixed gently, and collected by gravity flow at 4 °C. Subsequently, A280 of the washing liquid was measured. The wash step was repeated until the value of A280 is reaching at 0.001. Proteins were eluted with 20 ml elute buffer (50 mM NaH_2_PO_4_, 300 mM NaCl, 200 mM imidazole, 1 mM PMSF, pH 8.0). The eluates were concentrated by centrifugation and stored in a −80 °C refrigerator for future use. Depending on the condition of the specific experiment and the nature of the protein, different elution buffers could be utilized to dissolve the concentrated protein.

### Electrophoretic mobility shift assay

The probe was synthesized and labeled with biotin with specific primers (Table S3). HinK protein was purified from *E. coli* BL21 (DE3) containing recombinant plasmids pET28a+hinK. To assess the DNA-binding activity of HinK to the promoter of *pqsR*, EMSA was carried out using a Protein-DNA binding Interaction Assay Kite LightShift Chemiluminescent EMSA kit (Thermo Fisher Scientific, Basingstoke, United Kingdom). The binding reactions were incubated at 25 °C for 30 min, and then loaded on 5% (w/v) native polyacrylamide gels and ran at 90 V for 180 min. Samples were transferred to a nitrocellulose filter membrane (Hercules, CA, USA). The membrane, followed by UV crosslinking at 245 wavelengths, was incubated with the streptavidin-HRP conjugate. Images were captured with the Analytik-jena imaging system. For detecting the role of TssZ^R5A/R9A^ in HinK-DNA^PqsR^ probe complex, EMSA binding reactions were incubated at 25 °C for 30 min, and then loaded on 5% (w/v) native polyacrylamide gels and ran at 90 V for 180 min. Gel was stained with SYBR Safe staining for 30 min and photographed by analytik-jena UVPGelSolo benchUV 2 touch 26Xi system.

### Swarming motility assays

Swarming motility analysis was performed with the NB semisolid medium containing 8 g/L nutrient Broth No.2 (Oxoid), 5 g/L Difco bacto-agar and 5 g/L D-glucose (Sigma) as previously described (60). The swarming plates were allowed to dry on a super clean bench at room temperature for 30 min before use. Overnight cultures were washed with liquid LB medium and diluted to an OD_600_ of 1.0. 3 μl dilution was inoculated onto swarming plates. Plates were then incubated for 16 h at 37 °C.

### Pyocyanin production assay

Pyocyanin production was measured in Low-phosphate (LP) media as previously described (61). The bacterial strains were cultured in 5 ml LB at 37 °C with continuous shaking at 250 rpm. Overnight cultures were diluted to an OD_600_ of 0.4. 1 ml dilution was added into 20 ml fresh LP medium and cultured at 37 °C for 12 h. 1 ml culture was collected and centrifuged at 12,000 rpm for 1 min 750 μl supernatant mixed with 450 μl chloroform and centrifuged at 12,000 rpm for 1 min. The upper aqueous phase was removed carefully. 400 μl of lower liquid was mixed with 200 μl 0.2 N HCl and then centrifuged at 12,000 rpm for 1 min. The absorbance of the upper phase was measured at 520 nm. The pyocyanin concentration was determined by multiplying A520 of supernatant by 17.072.

### Bacterial killing assay

The method used for bacterial killing assay was modified from that described by (62). Appointed *P. aeruginosa* strains used in this assay were grown overnight at 37 °C in 3 ml TSB medium, and prey reporter strain *E. coli* DH5α was cultured in 3 ml LB supplemented with 1 mM IPTG, 50 μg/ml apramycin. After overnight cultured, each *P. aeruginosa* strain was diluted and measured OD_600_ = 2.0. 500 μl diluted *P. aeruginosa* strains collected in a sterile 1.5 ml tubes and washed three times. *E. coli* cells were washed with fresh LB three times and diluted to OD_600_ of 0.4. 400 μl of *E. coli* cell cultures mixed with 400 μl that of *P. aeruginosa* in 1.5 ml tubes. Mixtures were centrifuged at 12,000 rpm for 1 min. Supernatants were discarded. Cells were resuspended with 80 μl LB. Inoculate 10 μl of the mixtures as a spot on the LB plates at 37 °C for 5 h, which the bacterial killing is occurring. For qualitative observation of this assay, bacterial spots collected with a sterile loop from previous inoculated plates and resuspend each spot in 1.5 ml tubes containing 1 ml of TSB medium. Place the tubes on the shaker for 30 min to resuspend. Proceed for each resuspended sample to 16 folds serial dilution, following spread 100 μl of the serial dilution on the LB plates containing 40 μg/ml X-gal, 1 mM IPTG, 50 μg/ml apramycin in triplicate within a quadrant. Plates were placed in 37 °C overnight about 18 to 24 h, and subsequently count CFU of blue color *E. coli*.

## Data availability

All data are contained within the manuscript and supporting information.

## Supporting information

This article contains supporting information.

## Conflict of interest

The authors declare that they have no conflicts of interest with the contents of this article.
