# Supplementary material for: A PilZ domain protein interacts with the transcriptional regulator HinK to regulate type VI secretion system in Pseudomonas aeruginosa
Source: J Biol Chem. 2024 Feb 9;300(3):105741. doi: 10.1016/j.jbc.2024.105741 (PMC10912698; doi:10.1016/j.jbc.2024.105741)
Supplement: Supporting Information [file mmc1.docx]

**Supplementary material**

A PilZ Domain Protein Interacts with the Transcriptional Regulator HinK to Regulate Type VI Secretion System in *Pseudomonas aeruginosa*

Tianfang Cheng**^1^**, Qing Wei Cheang**^2^**, Linghui Xu**^1^**, Shuo Sheng**^1,3^**, Zhaoting Li**^1^**, Yu Shi**^1^**, Huiyan Zhang**^1^**, Li Mei Pang^2^, Ding Xiang Liu**^1^**, Liang Yang^2^, Zhao-Xun Liang**^2*^**, Junxia Wang**^1^**^*^

^1^Integrative Microbiology Research Centre, Guangdong Province Key Laboratory of Microbial Signals and Disease Control, South China Agricultural University, Guangzhou 510642, China.

^2^School of Biological Sciences, Nanyang Technological University, 60 Nanyang Drive, Singapore 637551, Singapore.

^3^Key Laboratory of Basic Pharmacology of the Ministry of Education, Joint International Research Laboratory of Ethnomedicine of the Ministry of Education and Key Laboratory of Basic Pharmacology of Guizhou Province, Zunyi Medical University, Zunyi, Guizhou, China

*Correspondence:

Zhao-Xun Liang

zxliang@ntu.edu.sg

Junxia Wang

[junxiawang@scau.edu.cn](mailto:junxiawang@scau.edu.cn)

**Content**

Figure S1. Expression of HSI-I gene cluster was downregulated in TssZ overexpression strain.

Figure S2. HinK binds to the promoter of *pqsR*.

Figure S3. Verification of mutants.

Table S1. Bacterial strains and yeast strains used in this study.

Table S2. Plasmids used in this study.

Table S3. Primers used in this study.

**
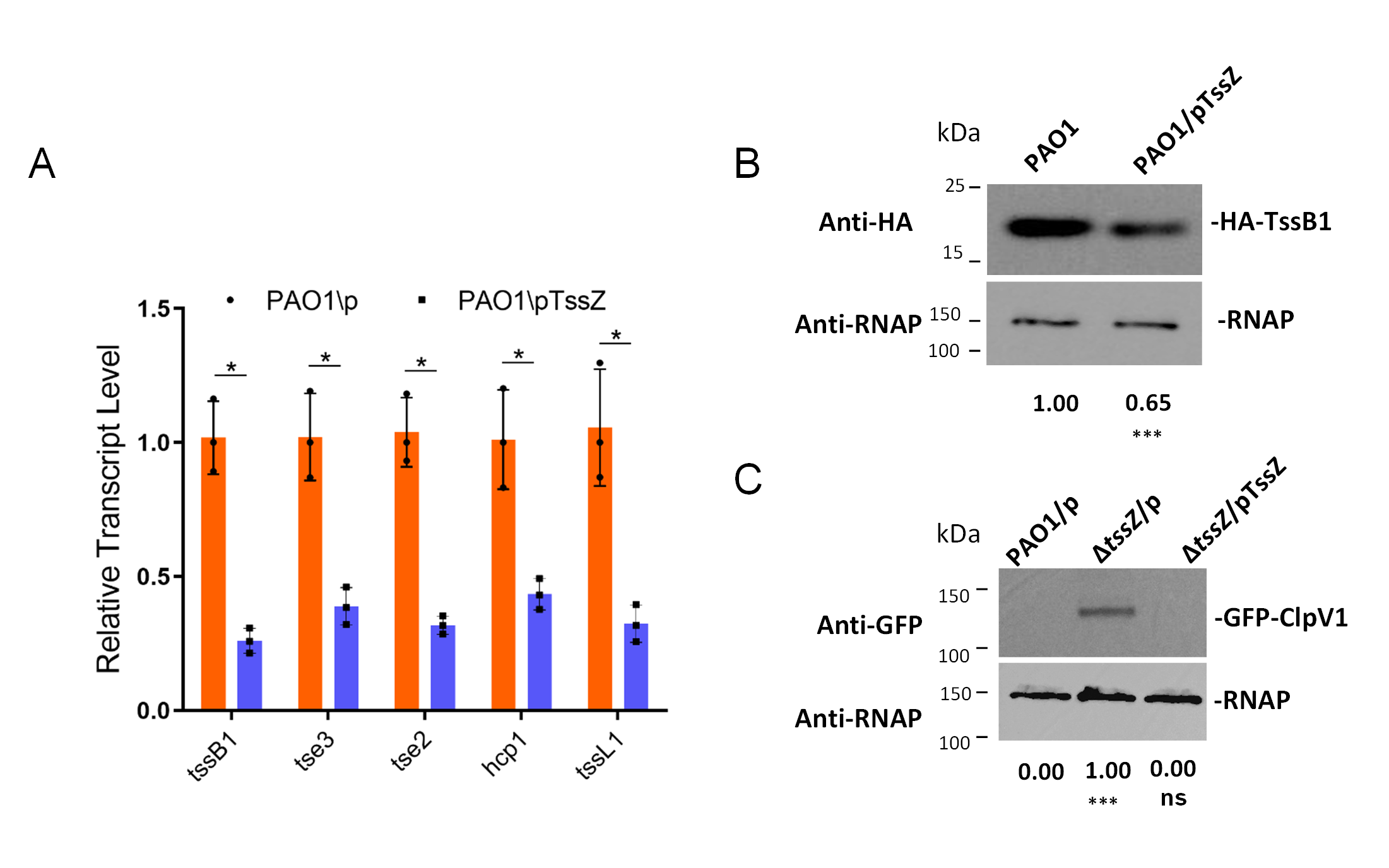
**

**Figure S1. Expression of HSI-I gene cluster was downregulated in the TssZ overexpressing strain.** (A) Transcription levels of the *tssB1*, *tse3*, *tse2*, *hcp1*, and *tssL1* in wild type PAO1 and TssZ overexpressing strains. RNA was extracted from strains cultured in M9 medium. The *rplu* gene was chosen as an internal reference gene. All experiments were repeated independently for three times and data are represented as the mean ± SD (n = 3). (B) The expression levels of TssB1-HA protein and ClpV1-GFP protein were detected in PAO1 with / without TssZ overexpression by Western blot*.* The expression level of RNA polymerase (RNAP) alpha subunit was used as an internal reference. Band intensities were determined using ImageJ software and the ratio of TssB1-HA band to RNAP band was calculated and indicated at the bottom of each sample’s respective lane. The ratio for the PAO1-pUCP18 control strain was used as a baseline of 1 and other ratios were normalized relative to it. All experiments were repeated independently for three times and data are represented as the mean ± SD (n = 3). (C) The expression levels of ClpV1-GFP protein were detected in PAO1, Δ*tssZ* with / without TssZ complement strains by Western blot*.* Similar to (B), RNA polymerase (RNAP) alpha subunit was used as an internal reference and the band intensity ratio of the GFP to RNAP was calculated for all samples. The Δ*tssZ* strain with pUCP18 was used as the control and the ratio of other samples were normalized relative to it. All experiments were repeated independently for three times and the standard error is indicated by the error bars. Student's *t*-test was used to compare between samples (* p < 0.05, ** p < 0.01, *** p < 0.001, n = 3).

**
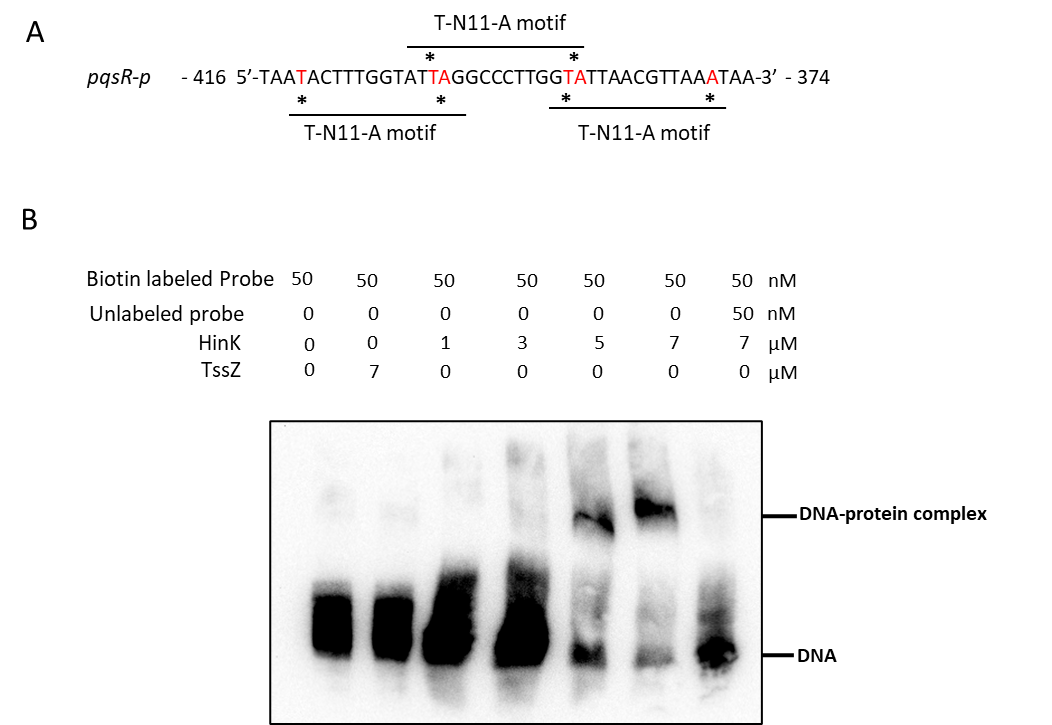
**

**Figure S2. HinK binds to the promoter of *pqsR*.** (A) Potential LTTR box (T-N11-A consensus motif) in the *pqsR* promoter (*pqsR-p*) are located -415 – -375 bp relative to the start codon. The conserved thymine and adenine bases that mark the start and end of the motif respectively is indicated by the asterisk and red font. (B) Electrophoretic Mobility Shift Assay (EMSA) demonstrates the binding of HinK to the promoter of *pqsR.*

*
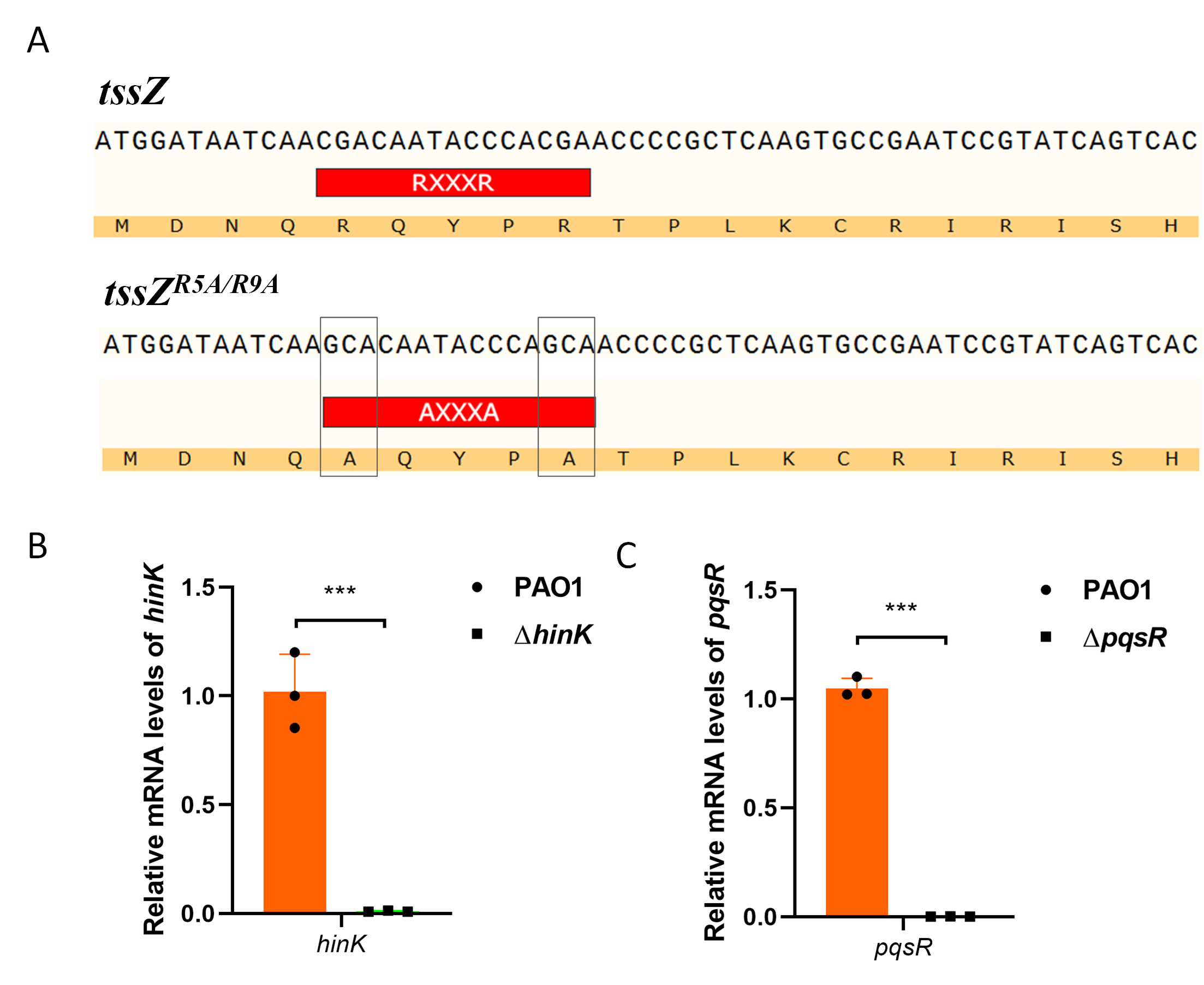
*

**Figure S3. The *tssZ^R5A/R9A^*, *hinK* and *pqsR* deletion mutants.** (A) DNA sequencing verified *tssZ* point mutation in *tssZ^R5A/R9A^* mutant. DNA fragment was amplified using the primes designed from upstream and downstream sequence of *tssZ* gene. TsingKe Company conduct sequencing work for DNA fragments. We display the 60 bp sequence (5’-3’) of tssZ and *tssZ^R5A/R9A^* here. Red box indicates RxxxR or AxxxA motif, black rectangle represented mutation site and letters in yellow box represent amino acid in these two sequences. (B) Transcription levels of *hinK* in the Δ*hinK* mutant strain. The *recA* gene was used as an internal reference gene. (C) Transcription levels of *pqsR* in the Δ*pqsR* mutant strain. The *recA* gene was used as an internal reference gene. All experiments were repeated independently for three times and data are represented as the mean± SD (n = 3). Student's t-test was used to analyze compare the results (* p < 0.05, ** p < 0.01, *** p < 0.001, n = 3).

**Table S1. Bacterial strains and yeast strains used in this study**

| **Strains** | **Relevant characteristic(s)** | **Source** |
| --- | --- | --- |
| *P. aeruginosa* |  |  |
| PAO1 | Wild type (parental strain) of *P. aeruginosa* | Laboratory preservation |
| *tssZ^R5A/R9A^* | *tssZ^R5A/R9A^* point mutant (Point mutation of TssZ to destroy the binding ability of TssZ to c-di-GMP) | Constructed in this study |
| Δ*hinK* | *hinK* knock-out mutant | Constructed in this study |
| Δ*pqsR* | *pqsR* knock-out mutant | Constructed in this study |
| *tssZ^R5A/R9A^*Δ*pqsR* | *tssZ^R5A/R9A^* point and *pqsR* knock-out double mutant | Constructed in this study |
| Δ*hinK*Δ*pqsR* | *HinK and pqsR* double-knock-out mutant | Constructed in this study |
| *Escherichia coli* |  |  |
| *E. coli* DH5α | *E. coli* cells for cloning the recombinant plasmid | Laboratory preservation |
| *E. coli* BL21(DE3) | *E. coli* cells for expression of heterologous protein | Laboratory preservation |
| *Saccharomyces cerevisiae* |  |  |
| Y2HGold | *S. Cerevisiae* cells for yeast two hybrid assay | (Coolaber, Beijing, China) |

**Table S2. Plasmids used in this study**

| **Plasmid** | **Relevant characteristic(s)** | **Source** |
| --- | --- | --- |
| pRK2013 | Helper plasmid used in triparental matings; Kana^r^ | Laboratory preservation |
| pK18 | Plasmid for replacement of *target gene*; Gm^r^ | Laboratory preservation |
| pK18-*tssZ^R5A/R9A^* | Plasmid carrying sequences of *tssZ^R5A/R9A^* for replacement of *tssZ*; Gm^r^ | Constructed in this study |
| pK18-*hinK* | Plasmid carrying homology arm sequences of *hinK* for removal of *hinK*; Gm^r^ | Constructed in this study |
| pK18-*pqsR* | Plasmid carrying homology arm sequences of *pqsR* for removal of *hinK*; Gm^r^ | Constructed in this study |
| pET28a+*tssZ* | Plasmid for expression of TssZ-6×His protein; Kana^r^ | Constructed in this study |
| pET28a+*tssZ^R5A/R9A^* | Plasmid for expression of TssZ^R5A/R9A^-6×His protein; Kana^r^ | Constructed in this study |
| pET28a+*hinK* | Plasmid for expression of HinK-6×His protein; Kana^r^ | Constructed in this study |
| pUCP18 | Plasmid as blank control; perform cb resistance in *P. aeruginosa,* amp resistance in *E. coli* | Laboratory preservation |
| pUCP18-*tssZ* | Plasmid for overexpression of *tssZ*; perform cb resistance in *P. aeruginosa,* amp resistance in *E. coli* | Constructed in this study |
| pUCP18-*pqsR* | Plasmid for overexpression of *pqsR*; perform cb resistance in *P. aeruginosa,* amp resistance in *E. coli* | Constructed in this study |
| pGBKT7-TssZ | Bait plasmid used in yeast two-hybrid screening; Kana^r^ | Constructed in this study |
| (*Continued on next page*) | | |

**Table S2. (*Continued from previous page*)**

| **Plasmid** | **Relevant characteristic(s)** | **Source** |
| --- | --- | --- |
| pGADT7-HinK | Prey plasmid for yeast two-hybrid analysis; Amp^r^ | Constructed in this study |
| pME6032 | Plasmid as blank control; Tc^r^ | Laboratory preservation |
| pME6032-*PA2133* | Plasmid for overexpression of PA2133 to decrease intracellular c-di-GMP; Tc^r^ | Laboratory preservation |
| pME6032-*wspR* | Plasmid for overexpression of WspR to increase intracellular c-di-GMP; Tc^r^ | Laboratory preservation |
| pBBR1-V5-HinK | Plasmid for Co-immunoprecipitation; Gmr | Constructed in this study |
| pBBR1-V5-HinK  -TssZ-HA | Plasmid for Co-immunoprecipitation; Gm^r^ | Constructed in this study |
| pBBR1-V5-HinK  -TssZ^R5A/R9A^-HA | Plasmid for Co-immunoprecipitation; Gm^r^ | Constructed in this study |
| pPROBE-*p-tssA1-GFP* | *p-tssA1-GFP* transcriptional fusion plasmid to measure expression of TssA1(first gene of the HSI-I operon), Gm^r^ | Constructed in this study |

**Table S3. Primers used in this study**

| **Primer Name** | **Sequence (5'-3')** | **Function** |
| --- | --- | --- |
| *pBBR1-F* | CTGTTTCTCCATACCCGTT | PCR analysis for plasmids |
| *pBBR1-R* | GTAAAACGACGGCCAGT |  |
| *pME6032-F* | ACTTCACTGACACCCTCATCAGTG |  |
| *pME6032-R* | GCAAGCTGATCCGGGCTTAT |  |
| *pET28a-F* | GGGAATTGTGAGCGGATAAC |  |
| *pET28a-R* | CTTCCTTTCGGGCTTTGTTA |  |
| *pUCP18-F* | CCAGCTGCGAAAGTGGTTTCAAC |  |
| *pUCP18-R* | CAATACGCAAACCGCCTCTCCC |  |
| *pK18-F* | TGCTTCCGGCTCGTATG |  |
| *pK18-R* | GCGAAAGGGGGATGTGC |  |
| *pPROBE-F* | GCCATAAACTGCCAGGAATTG |  |
| *pPROBE-R* | TTGTGCCCATTAACATCACC |  |
| *PtssA1_F* | GAATTGGGGATCGGAAGCTTTGCTACTCCTTGCATTGCCAG | *tssA1* promoter for GFP transcriptional fusion assay |
| *PtssA1_R* | TTAGTTAGTTAGGGAATTCGGTGACGATCTCCCTATCATCG |  |
| *EMSA-PpqsR_F* | TAGGTCGCGCCAGGGCCAT | *pqsR* promoter probe for EMSA assay |
| *EMSA-PpqsR_R* | GAGGGATGACGGCTCTGTTTC |  |
| *qrecA_F* | CGAGACCGTCGGCAAATAC | qRT-PCR for reference gene *recA* |
| *qrecA_R* | GCACCTGGTCGATGTGAAA |  |
| *qrhlA_F* | CGAGACCGTCGGCAAATAC | qRT-PCR for *rhlA* |
| *qrhlA_R* | GCACCTGGTCGATGTGAAA |  |
| (*Continued on next page*) | | |

**Table S3. (*Continued from previous page*)**

| **Primer Name** | **Sequence (5'-3')** | **Function** |
| --- | --- | --- |
| *qrhlB_F* | TGTCACAACCGCACAGTATC | qRT-PCR for *rhlB* |
| *qrhlB_R* | CTTCAGCCATCGAGCATCC |  |
| *qrhlC_F* | GTGCTGGTGGTACTGTTCAA | qRT-PCR for *rhlC* |
| *qrhlC_R* | GTTGTCGACGGCAAGGAA |  |
| *qphzA1_F* | GCTATTGCGAGAACCACTACA | qRT-PCR for *phzA1* |
| *qphzA1_R* | CAATGCACGCAGTTTCTGTATC |  |
| *qphzM_F* | CTGCTGCGCGTAATTTGATAC | qRT-PCR for *phzM* |
| *qphzM_R* | TCGATCCCGCTCTCGAT |  |
| *qtssB1_F* | AGGTCGATTCGCTGAACAAG | qRT-PCR for *tssB1* |
| *qtssB1_R* | CTTGATCGCCTTCATGATCATTTC |  |
| *qclpV1_F* | GTGCTGGTGGTACTGTTCAA | qRT-PCR for *clpV1* |
| *qclpV1_R* | GTTGTCGACGGCAAGGAA |  |
| *qhcp1_F* | AGGACCTGTCGTTCACCAA | qRT-PCR for *hcp1* |
| *qhcp1_R* | ATAGTGCTTGCCGCTGGA |  |
